# Arsenotoxicidad aguda experimental en ratones Balb/c: marcadores orgánicos y compromiso esplénico

**DOI:** 10.7705/biomedica.5485

**Published:** 2020-10-01

**Authors:** Alejandra Mariel Canalis, Roberto Daniel Pérez, Gisele Evangelina Falchini, Elio Andrés Soria

**Affiliations:** 1 Escuela de Nutrición, Facultad de Ciencias Médicas, Universidad Nacional de Córdoba, Córdoba, Argentina; 2 Consejo Nacional de Investigaciones Científicas y Técnicas, CONICET, Instituto de Investigaciones en Ciencias de la Salud, Córdoba, Argentina; ^3^Facultad de Matemáticas, Astronomía y Física, Universidad Nacional de Córdoba, Córdoba, Argentina ^4^Consejo Nacional de Investigaciones Científicas y Técnicas, CONICET, Instituto de Física “Enrique Gaviola”, Córdoba, Argentina; 5 Cátedra de Biología Celular, Histología y Embriología, Facultad de Ciencias Médicas, Universidad Nacional de Córdoba, Córdoba, Argentina

**Keywords:** arsénico, toxicidad aguda, estrés oxidativo, sistema inmunológico, ratones, Arsenic, acute toxicity, oxidative stress, immune system, mice

## Abstract

**Introducción:**

El arsénico es un tóxico ambiental ampliamente diseminado en todo el mundo. En hombres y animales, diversos órganos y tejidos son blancos de sus efectos deletéreos, entre ellos, el los del sistema inmunológico.

**Objetivo:**

Determinar la intoxicación aguda por arsénico en tejidos y células diana de ratones Balb/c *in vivo*.

**Materiales y métodos:**

Se aplicó una inyección intraperitoneal de 9,5 o 19 mg/kg de arsenito de sodio (NaAsO_2_) o un volumen equivalente de solución fisiológica como control, en ratones Balb/c con 3 por cada grupo experimental. Tras media hora, los animales fueron sacrificados y se extrajeron bazos, timos, hígados, riñones y sangre. En cada muestra, se determinó la concentración de arsénico, polifenoles y hierro, y también, se evaluaron marcadores oxidativos, como peróxidos, productos avanzados de oxidación proteica y grupos sulfhidrilos libres. En los esplenocitos obtenidos del bazo, se determinaron la viabilidad celular y el potencial mitocondrial.

**Resultados:**

La dosis aguda inyectada de NaAsO_2_ redujo la función mitocondrial de los esplenocitos, lo que derivó en muerte celular. La presencia confirmada de arsénico en las muestras de bazo y la citotoxicidad resultante, produjeron disminución de los polifenoles y de los grupos sulfhidrilos libres, y alteraron el contenido y la distribución del hierro, pero no se aumentó la producción de peróxidos.

**Conclusión:**

Estos hallazgos aportan evidencia científica sobre los cambios en biomarcadores involucrados en la inmunotoxicidad del arsénico y ofrecen, además, una metodología para ensayar potenciales tratamientos frente a la acción deletérea de este compuesto en el sistema inmunológico.

## Abstract (en)

**Introduction:**

Arsenic is an environmental toxic present worldwide. In men and animals, various organs and tissues are targets of its deleterious effects including those of the immune system.

**Objective:**

To determine acute arsenic toxicity in tissues and target cells of Balb/c mice using an *in vivo* methodology.

**Materials and methods:**

We injected Balb/c mice intraperitoneally with 9.5 or 19 mg/ kg of sodium arsenite (NaAsO_2_), or an equivalent volume of physiological solution as a control (with 3 per experimental group). After 30 minutes, the animals were sacrificed to obtain spleen, thymus, liver, kidneys, and blood. We determined arsenic, polyphenols, and iron concentrations in each sample and we evaluated the oxidative markers (peroxides, advanced products of protein oxidation, and free sulfhydryl groups). In splenocytes from the spleen, cell viability and mitochondrial potential were also determined.

**Results:**

The exposure to an acute dose of NaAsO_2_ reduced the mitochondrial function of splenocytes, which resulted in cell death. Simultaneously, the confirmed presence of arsenic in spleen samples and the resulting cytotoxicity occurred with a decrease in polyphenols, free sulfhydryl groups, and an alteration in the content and distribution of iron, but did not increase the production of peroxides.

**Conclusion:**

These findings provide scientific evidence about changes occurring in biomarkers involved in the immunotoxicity of arsenic and offer a methodology for testing possible treatments against the deleterious action of this compound on the immune system.

Elio Andrés Soria, Instituto de Investigaciones en Ciencias de la Salud, Ciudad Universitaria, Córdoba 5014, Argentina

Los compuestos derivados del arsénico son agentes químicos con una elevada toxicidad para el ser humano. Sus fuentes de procedencia son diversas y estos compuestos pueden ingresar al organismo por las vías respiratorias o la piel y, con mayor frecuencia, por su ingestión accidental, o la de agua o alimentos contaminados (1,2).

El arsénico está presente en el agua como un oxianión y tiene dos estados de oxidación mayoritarios en la naturaleza: el arsenito o As^+3^ y el arseniato o As^+5^. Estas dos especies están en la fracción soluble del arsénico, por lo que la filtración de las muestras con un filtro de membrana de 0,45 pm permite separar el arsénico en partículas del arsénico disuelto en el agua.

En diversos estudios se han determinado los mecanismos de toxicidad del arsénico en sus distintos estados de oxidación, los cuales producen efectos deletéreos en los órganos y tejidos hemolinfáticos (3). En este sentido, se sabe que la toxicidad del arsenito o As^+3^ es varias veces mayor que la del arseniato o As^+5^ debido a su mayor reactividad y a que los compuestos arsenicales de naturaleza inorgánica poseen un poder tóxico superior al de los derivados orgánicos de este elemento (4). No obstante, todos los derivados del arsénico constituyen poderosos agentes inmunotóxicos causantes comprobados de enfermedades infecciosas y de tumores y otras condiciones crónicas, pues promueven inmunodeficiencia secundaria con compromiso de las defensas del organismo (5).

Actualmente, se sabe que entre 60 y 100 millones de personas en todo el mundo están expuestas a este contaminante y son vulnerables a sus efectos tóxicos (6), lo que lo convierte en un serio problema de salud pública en numerosos países, entre ellos la República Argentina, donde se requieren investigaciones que aporten datos significativos para avanzar en el manejo y la solución de este problema.

En este contexto, el presente estudio se centró en determinar los efectos del arsénico en los biomarcadores involucrados en su inmunotoxicidad y, en consecuencia, en el desarrollo de una metodología adecuada para el ensayo de potenciales protectores frente a su acción deletérea.

## Materiales y métodos

### Condiciones experimentales

Se emplearon ratones Balb/c machos de ocho semanas de edad, que se mantuvieron bajo las condiciones estándar de bioterio con alimentación e hidratación *ad libitum*, utilizando productos comerciales (200 ± 13 g/kg/ día, Cargill™ SACI, Argentina) y agua potable (150 ± 10 ml/kg/día; Aguas Cordobesas SA, Argentina), hasta el momento de la intervención.

Los animales fueron testeados y sacrificados conforme a la metodología evaluada y aprobada por el Comité Institucional para el Cuidado y Uso de Animales de Laboratorio (CICUAL) de Córdoba, Argentina, el cual acoge y reglamenta los principios y normas internacionales para el uso de animales en investigación (en este estudio: UNC-FCM-SECYT-CICUAL-2014-09-02). Según dichas normas, los animales se anestesiaron por inhalación de isoflurano (Piramal Healthcare, Reino Unido) para su sacrificio y, luego, se procedió a su sangrado total mediante punción cardíaca.

Se conformaron: un grupo de 3 ratones con 9,5 mg/kg, otro grupo de 3 ratones con 19 mg/kg y otro grupo control de 3 ratones con 100 pl de solución salina (N=9), que recibieron una inyección intraperitoneal empleando jeringas de 1 ml (0,50 x 15 mm - 25 G 5/88”-). Para esto, se usó arsenito de sodio (NaAsO_2_, forma molecular disponible de arsénico trivalente con mayor toxicidad) (Laboratorio Anedra, Argentina), en 100 pl de solución acuosa [dosis letal (DL5_0_) intraperitoneal en ratones: 19,0 mg/kg - 46,3 pmol/kg de peso corporal) (7).

El sacrificio de los animales y su posterior autopsia se llevaron a cabo una vez transcurridos 30 minutos desde la administración de la inyección intraperitoneal, tiempo durante el cual se les mantuvo con hidratación oral *ad libitum* y se observó la evolución clínica de cada individuo.

Se obtuvo sangre por punción cardíaca de los animales anestesiados y, luego, se extrajeron el timo, el bazo, el hígado y el riñón, para su posterior procesado y análisis.

El bazo se dividió en dos mitades, una de las cuales se empleó para el cultivo primario de los esplenocitos. La otra mitad, junto con los demás órganos, se homogeneizó mecánicamente en 2 ml de solución salina isotónica.

Cada muestra líquida así obtenida se dividió en dos alícuotas previa medición del contenido proteico empleando un estuche comercial (Wiener Lab Group, Argentina) para estandarizar las variables que posteriormente se estudiaron. Así, los homogenatos de tejidos se emplearon en la determinación de arsénico, hierro, peróxidos, productos avanzados de oxidación proteica y grupos sulfhidrilos libres.

Por otra parte, la fracción restante fue tratada con metanol y ácido tricloroacético al 50% (relación respectiva de volúmenes 4:6:0,5) a 50 °C durante 30 minutos (Cicarelli, Argentina), con el fin de obtener un sobrenadante por centrifugación a 10.000*g* durante un minuto para dosificar los polifenoles. Todas las muestras se conservaron a -18 °C hasta el momento de su análisis.

### Cultivo primario de esplenocitos

Bajo condiciones de máxima asepsia, se disgregó mecánicamente cada muestra esplénica en 2 ml de solución hemolítica. La suspensión celular obtenida se recogió en un tubo cónico estéril, se centrifugó para recuperar el precipitado y se resuspendió en 10 ml de medio de cultivo RPMI 1640 sin rojo fenol con 10% de suero fetal bovino, 2 g/L de bicarbonato de sodio, 10 mg/L de ciprofloxacina y 3,25 pl/L de mercaptoetanol.

Por último, se contabilizaron las células en una cámara de Neubauer y los resultados se expresaron como células/pl. Las suspensiones celulares se mantuvieron a 37 °C en ambiente estéril con 5% de CO_2_ hasta su análisis.

### Determinaciones

*Arsénico.* Se determinó su presencia en muestras de bazo y riñón (este último por su rol en la excreción de este mineral) mediante fluorescencia de rayos X. Para ello, se depositaron 30 pl del homogenato de cada muestra agregando un estándar interno de cloruro de estroncio sobre una película de celulosa de 0,1 mm de espesor. Posteriormente, cada una de las muestras fue irradiada con rayos X de 17,44 keV seleccionados mediante un monocromador de cristal en un tubo de rayos X de 3 kW de potencia con foco lineal de 12 mm x 0,4 mm y un ánodo de molibdeno (modelo PW2275/20, Philips, Países Bajos).

La radiación característica emitida por el arsénico se registró con un detector de estado sólido dispersivo en energía de cristal de silicio (modelo XT, marca MOXTEK Inc., Estados Unidos). La preparación de la muestra delgada combinada con la excitación monocromática permitió minimizar el ruido espectral, mejorando considerablemente el límite de detección de la técnica y alcanzando los 0,2 ppm p/p para el As (8).

### Viabilidad celular

La metodología y las condiciones óptimas de reacción se ensayaron y se establecieron previamente. Posteriormente, las suspensiones celulares se incubaron en una placa de 96 pozos, en oscuridad y a 37 °C, con una solución de 0,05 mg/ml de resazurina en proporción de 1:100 v/v. Pasadas cinco horas, se procedió a medir la absorbancia a 600 nm usando un multilector de microplacas Multi-GloMax™ (Promega Corp., USA) y se calcularon los niveles de viabilidad celular como porcentaje de absorbancia con respecto al grupo de control (%) luego de estandarizar los valores obtenidos por el número de células contenido en cada suspensión celular (9).

### Polifenoles

Mediante la técnica de Folin-Ciocalteu, se incorporaron solución de Folin 2 N, agua destilada y solución saturada de bicarbonato de sodio a los sobrenadantes ácido-metanólicos de las muestras, los cuales se habían sembrado previamente en una placa de 96 pozos en proporción de 1:6:2:1 v/v/v/v. La mezcla resultante se incubó en oscuridad durante 30 minutos a 37 °C, tras lo cual se midió la absorbancia a 750 nm y se calculó el contenido de polifenoles por interpolación de las lecturas obtenidas con una curva estándar de ácido gálico (0-0,1 mg/ ml) expresada en mg de proteína (EAG pg/mg de proteína, proteína, con EAG como equivalentes de ácido gálico) (10).

### Hierro

Este se determinó en los homogenatos de las muestras midiendo por espectrofotometría a 570-640 nm la formación de un complejo azul-violáceo a partir de los cationes ferrosos con 3-(2-piridil)-5,6-bis-2(sulfofuril)-1,2,3-triazina (fereno) (Ferroquant, GT Lab, Argentina). Los resultados se expresaron como pg de hierro por mg de proteína, para lo cual se empleó como estándar la solución de sales ferrosas de 100 pg/dl provista por el estuche comercial (11).

### Peróxidos acuosos y lipídicos

Cada muestra se sometió a reacción con la solución cromógena (1:10 v/v) y se incubó durante 30 minutos a temperatura ambiente. Para el peróxido acuoso, dicha solución consistió en 25 mM de sulfato de amonio ferroso en 2,5 M de ácido sulfúrico reconstituido con 100 mM de sorbitol y 125 pM de naranja de xilenol. Para el peróxido lipídico), el sulfato de amonio ferroso se reconstituyó con 4 mM de hidroxitolueno butilado y 125 pM de naranja de xilenol en metanol al 90% (Sigma-Aldrich Co., USA). Las concentraciones de ambos peróxidos se calcularon como porcentajes de absorbancia a 540 nm con respecto al grupo de control, tras restar el blanco correspondiente y estandarizar según el contenido proteico (12).

### Productos avanzados de oxidación proteica

Esto se midieron mediante espectrofotometría a 340 nm en condiciones acídicas y en presencia de yoduro de potasio, siguiendo la reacción de yoduro a diyodo que provocan los productos avanzados de oxidación proteica y utilizando como patrón de referencia la cloramina T. Para ello, se colocó la muestra en una placa de 96 pozos con 0,015 M de solución PBS, 16 M de yoduro de potasio (IK 1) y ácido acético en una relación de 4:16:1:2. La concentración de estos productos se calculó con base en la ecuación de la curva del estándar y se expresó como mg equivalentes de cloramina T por mg de proteína (13).

### Grupos sulfhidrilos libres

Para determinarlos en las muestras, se utilizó el reactivo de Ellman, 5, 5’-ditio-bis- (ácido 2-nitrobenzoico) (Sigma-Aldrich Co., USA), también conocido como DTNB. La técnica necesitó ser desarrollada y puesta a punto, para lo cual se preparó una solución madre de 10 mM de DTNB, empleando DMSO como solvente que luego se diluyó 100 veces con tris-HCl 0,1M pH 7,5 para obtener una solución de 0,1 mM cada vez que fue necesario.

De esta manera, 190 pl de la solución diluida se incorporaron a los homogenatos de muestras (10 pl) en una placa de 96 pozos y se incubaron durante una hora a temperatura ambiente antes de medir la absorbancia en el espectrofotómetro (412 nm). Los grupos sulfhidrilo se calcularon como mg de N-acetilcisteína por mg de proteína, para lo cual se restaron los blancos correspondientes a las lecturas obtenidas y se interpolaron los valores estandarizados por proteínas contenidas en la muestra con una curva del estándar (R^2^>0,97) realizada con 0,032- 1,02 pg de N-acetilcisteína (Sigma- Aldrich Co., USA) (7).

### Determinación del potencial mitocondrial

Se incubaron las muestras a 37 °C durante 15 minutos bajo condiciones de oscuridad en 148 pl de suspensión celular con 2 pl de reactivo de yoduro de 3,3'dihexiloxacarbocianina (Sigma-Aldrich Co., USA) preparado en dimetilsulfóxido (concentración final del fluoróforo de 40 nM) para, luego, leer la absorbancia a 485 nm. Tras estandarizar por conteo celular, los resultados se expresaron como porcentaje de área con respecto al grupo de control.

### Análisis estadístico

Los resultados obtenidos a partir de las distintas determinaciones se expresaron como media ± error estándar con el programa estadístico InfoStat 2018 (estadística descriptiva). Para establecer el efecto en las muestras animales, se empleó un análisis de varianza seguido por la prueba de Fisher (p<0,05) (14).

## Resultados

### Acumulación tisular de arsénico

Considerando los límites de detección de la metodología aplicada, la presencia de arsénico se confirmó en las muestras de bazo y riñón de los animales tratados con NaAsO_2_ (>0,2 ppm p/p), en tanto que, en aquellas del grupo de control, se determinaron su ausencia o sus valores no detectables (<0,2 ppm p/p). Estos hallazgos confirman la acumulación de arsénico en los grupos inyectados con NaAsO_2_.

### Disminución de la viabilidad celular

**Figura 1:**
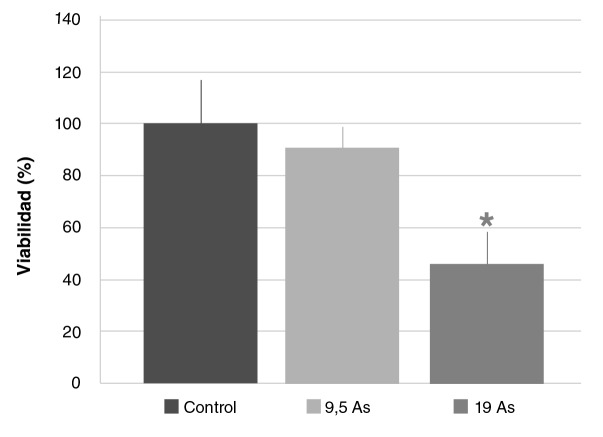
Viabilidad de esplenocitos obtenidos por cultivo primario de bazo de ratones Balb/c sometidos a las siguientes condiciones experimentales: 0 mg/kg de NaAsO_2_ (control), 9,5 mg/kg de NaAsO_2_ intraperitoneal (9,5 As), 19 mg/kg de NaAsO_2_ intraperitoneal (19 As). Los valores se expresan como medias (% de área según control) ± EE (n>3).

El análisis de los datos por regresión lineal indicó un descenso de la viabilidad celular dependiente de la concentración de arsénico (R2=0,7; p<0,0001), siendo significativa estadísticamente la reacción ante la DL_50_ intraperitoneal (19 mg/kg de NaAsO_2_) comparada con la del control (46,95%; p<0,05) (figura 1).

Según los resultados obtenidos, se hicieron las siguientes cuantificaciones en las muestras de bazo y timo del grupo de control y las del grupo que recibió la DL_50_ de 19 mg/kg de NaAsO_2_ intraperitoneal. Posteriormente, las pruebas se repitieron en las muestras de hígado y riñón de los mismos grupos.

### Compromiso de los polifenoles biodisponibles

**Figura 2:**
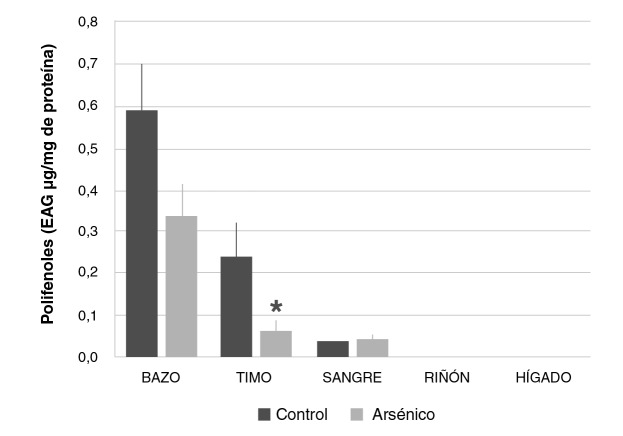
Concentración de polifenoles en bazo, timo, sangre, riñón e hígado de ratones Balb/c sometidos a las siguientes condiciones experimentales: 0 mg/kg de NaAsO_2_ (control), 19 mg/ kg de NaAsO2 intraperitoneal (arsénico). Los valores se expresan como medias (EAG pg/mg de proteína) ± EE (n>3).

La concentración de polifenoles dosificada en los bazos (0,33 ± 0,07 EAG pg/mg de proteína) y timos (0,06 ± 0,03 EAG pg/mg de proteína) del grupo que recibió arsénico fue inferior comparada con la del grupo control (0,59 ± 0,11 EAG pg/mg de proteína), verificándose una depleción en la biodisponibilidad de estos antioxidantes provenientes de la dieta, la cual resultó estadísticamente significativa en el timo. Por otra parte, no se observaron diferencias de la sangre y los órganos metabólicos entre los grupos experimentales (figura 2). Estos hallazgos demuestran un compromiso de la biodisponibilidad de los polifenoles por la incorporación de arsénico al organismo.

### Reducción variable del hierro tisular

**Figura 3:**
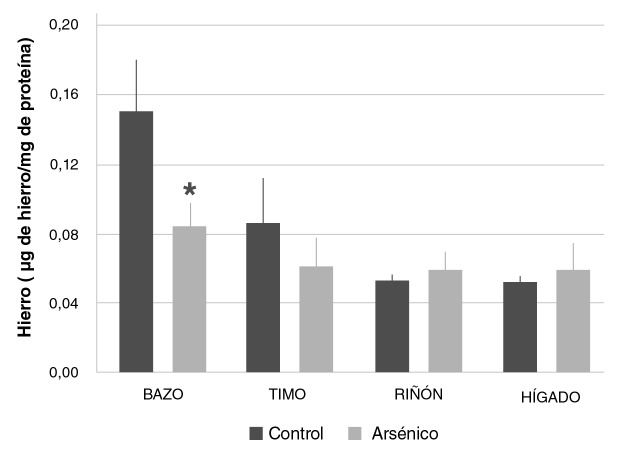
Concentración de hierro en bazo, timo, riñón e hígado de ratones Balb/c sometidos a las siguientes condiciones experimentales: 0 mg/kg de NaAsO_2_ (control), 19 mg/kg de NaAsO_2_ intraperitoneal (arsénico). Los valores se expresan como medias (pg de hierro/mg de proteína) ± EE (n>3).

La inyección intraperitoneal de la DL_50_ de arsenito de sodio condujo a una reducción significativa de la concentración total de hierro en el bazo (0,086 ± 0,012 pg de hierro/mg de proteína; p<0,05), comparada con la del control (0,150 ± 0,030 pg de hierro/mg de proteína), lo que da cuenta de una alteración mediada por el arsénico en el contenido y la distribución del hierro. No se observaron variaciones en la concentración del metal medido en los timos, los riñones y los hígados de los animales de los dos grupos experimentales comparados (figura 3).

### Oxidación renal sin compromiso de otros órganos

**Figura 4:**
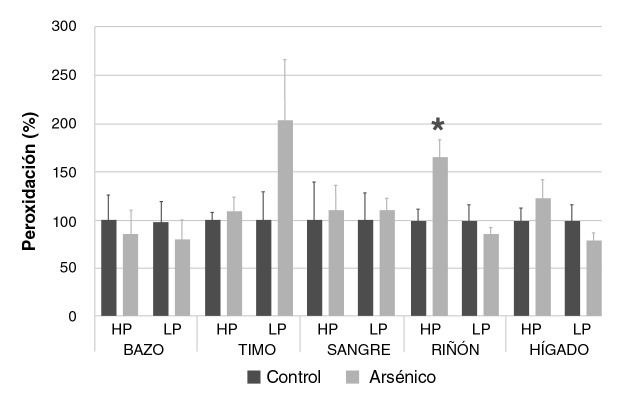
Formación de hidroperóxidos y lipoperóxidos de bazo, timo, sangre, riñón e hígado de ratones Balb/c sometidos a las siguientes condiciones experimentales: 0 mg/kg de NaAsO_2_ (control), 19 mg/kg de NaAsO_2_ lipoperóxidos (arsénico). Los valores se expresan como medias (% de área según control) ± EE (n>3).

El análisis de los resultados no evidenció diferencias significativas en la producción de peróxidos acuosos y lipídicos entre el grupo control y aquel con arsénico. Solo en los riñones del grupo con arsénico se observó un incremento en la producción de peróxido acuoso del 165,85% con respecto al control (p<0,05) (figura 4).

**Figura 5:**
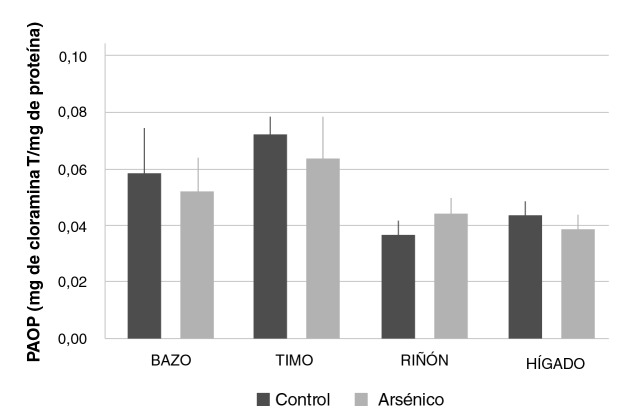
Formación de productos avanzados de oxidación proteica en bazo, timo, riñón e hígado de ratones Balb/c sometidos a las siguientes condiciones experimentales: 0 mg/kg de NaAsO_2_ (control), 19 mg/kg de NaAsO_2_ intraperitoneal (arsénico). Los valores se expresan como medias (mg de cloramina T/mg de proteína) ± EE (n>3).

Estos valores no evidenciaron diferencias significativas al evaluar y comparar los grupos de control y con arsénico en cuanto a la formación de productos avanzados de oxidación proteica que tiene lugar como resultado de la oxidación de macromoléculas de naturaleza proteica (figura 5).

La evaluación simultánea de estos datos evidenció que la dosis aguda empleada solo indujo estrés oxidativo en el riñón, por lo que no sería esta la causa del descenso de viabilidad observado en los esplenocitos.

### Efecto diferencial en los grupos sulfhidrilos libres

**Figura 6:**
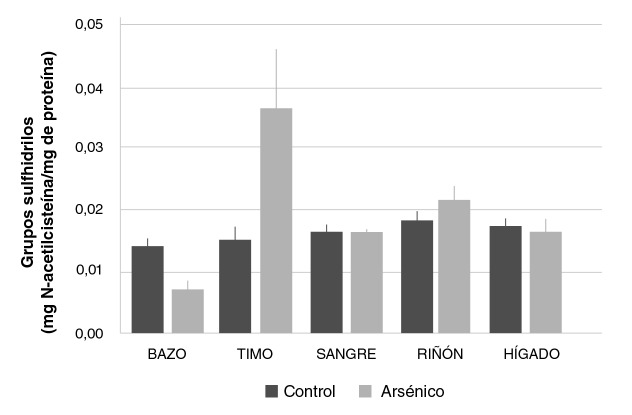
Concentración de grupos sulfhidrilos en bazo, timo, sangre, riñón e hígado de ratones Balb/c sometidos a las siguientes condiciones experimentales: 0 mg/kg de NaAsO_2_ (control), 19 mg/kg de NaAsO_2_ ip (arsénico). Los valores se expresan como medias (mg de N-acetilcisteína/ mg de proteína) ± EE (n>3).

Tras la inyección intraperitoneal de arsenito de sodio, la concentración de sulfhidrilos libres en el bazo tendió a disminuir (0,0072 ± 0,0014 mg de N-acetilcisteína/mg de proteína) con respecto al control (0,0142 ± 0,0014 mg de N-acetilcisteína/mg de proteína), aunque no representó una reducción estadísticamente significativa. En el timo, por el contrario, se presentó una tendencia al incremento de la concentración de los sulfhidrilos libres en las muestras obtenidas del grupo con arsénico (0,037 ± 0,009 mg de N-acetilcisteína/mg de proteína) comparado con el control (0,015 ± 0,002 mg de N-acetilcisteína/mg de proteína). Además, en las muestras de sangre, riñones e hígados, no se observaron variaciones significativas entre los resultados de ambos grupos (figura 6). Los hallazgos tras la medición de este biomarcador evidenciaron una interacción del arsénico administrado con los sulfhidrilos libres disponibles en el bazo y el timo.

### Disminución del potencial mitocondrial

Se observó una reducción del 23,08% en el potencial mitocondrial de los esplenocitos obtenidos de los animales que recibieron la DL_50_ de arsenito de sodio. En este sentido, el análisis estadístico de los datos disponibles determinó un efecto inverso o negativo dependiente de la dosis del arsénico (p=0,0632), lo que explica la caída en la función mitocondrial correlacionada con el incremento de este metal en el organismo.

## Discusión

Tras el análisis de los resultados obtenidos, pudo confirmarse la presencia de arsénico en las muestras de bazo y riñón de los animales inyectados con arsenito de sodio y su ausencia en los mismos tejidos provenientes del grupo de control, lo cual confirma que estos son acumuladores de compuestos arsenicales (15,16). Además, la presencia de arsénico en el bazo tras la administración de arsenito de sodio condujo a una pérdida de la viabilidad de los esplenocitos dependiente de la dosis, lo que confirma que son células diana de la inducción de muerte celular por los compuestos arsenicales (17).

Posteriormente, se midieron las concentraciones de hierro y polifenoles en las muestras orgánicas obtenidas de los animales experimentales y, al analizar la concentración de polifenoles, se observó una disminución tisular de estos compuestos en el bazo y el timo de los animales tratados con arsénico. Esto indica un posible agotamiento de los polifenoles provenientes de la dieta cuando los animales son tratados con el arsenito de sodio (18). Se encontraron resultados análogos en el bazo tras determinar la concentración de hierro, lo que corroboró la disminución de los niveles de este mineral con la administración de arsénico, lo cual podría explicarse por el desplazamiento del hierro hacia la corriente sanguínea o el espacio intersticial movilizado por la capacidad de lisis del arsénico (19). En este sentido, otros autores han reportado el efecto directo que tiene la bioacumulación de arsénico en el contenido y la distribución del hierro en los tejidos hemolinfáticos e, indirectamente, en la homeostasis de otros metales como el cobre, el cinc y el manganeso (20,21), lo cual compromete las reacciones enzimáticas de las células y contribuye a la inmunodeficiencia inducida por arsénico.

Además, se midió la formación de peróxidos acuoso y lipídico, y de productos avanzados de oxidación proteica en los tejidos de ambos grupos, y se encontró que la dosis de arsenito de sodio solo resultó en un aumento del peróxido acuoso en el riñón. Con la dosis de arsenito inyectada, se constató que la inducción de estrés oxidativo no sería la vía involucrada en la disminución de la viabilidad de los esplenocitos y, en una primera aproximación, los resultados indican una muerte celular temprana, sin variación en las mediciones de la oxidorreducción (22).

Por otra parte, con la administración de arsénico, la concentración de grupos sulfhidrilos en el bazo tendió a disminuir en comparación con el control, lo que se vio confirmado por la capacidad de los arsenicales trivalentes de interactuar con los grupos tioles de péptidos y proteínas, tales como el glutatión celular (GSH) y diversas enzimas y cofactores, y de inhibir su acción (23). No obstante, este efecto no se observó en el timo, donde la concentración de sulfhidrilos libres se elevó en el grupo con arsénico, probablemente como resultado de una diferenciación en el proceso de biotransformación que tiene lugar en ambos tejidos, a partir del cual el ácido metilarsónico se concentra en el timo, según lo observado por Xu, *et al.*; estos autores describen aumento de la capacidad de este metabolito para inducir genotoxicidad y apoptosis en dicho tejido (24), aunque se diferencia del arsénico trivalente en su selectividad por la interacción con los grupos tioles (25,26).

Por último, se observó una reducción en el potencial de la membrana mitocondrial de los esplenocitos obtenidos del grupo con arsénico en comparación con el de control. En este sentido, en varios estudios se plantea que el arsénico es un poderoso tóxico para la mitocondria con base en su capacidad de inducir apoptosis a partir de la acción directa sobre la cadena respiratoria mitocondrial y el poro de transición mitocondrial (mPTP) (27), lo cual aumenta la permeabilidad de las membranas interna y externa de la mitocondria, la liberación del citocromo C y la activación de las caspasas 9 y 3 (28). Consecuentemente, este evento deriva en una disminución del ATP y en la muerte celular (29), lo cual también explica la caída de la viabilidad observada en el presente trabajo.

En conjunto, los resultados brindan evidencia fehaciente de que la administración aguda en monodosis de arsenito de sodio reduce la función mitocondrial de los esplenocitos, lo que modularía la muerte celular, revalidando así la reconocida actividad citotóxica de los compuestos arsenicales (30,31). Además, se demostró que los mecanismos de toxicidad de esta sustancia involucran disminución en los niveles de polifenoles, y alteración en el contenido de sulfhidrilos libres y la distribución de hierro en perjuicio de los tejidos estudiados.

Los hallazgos del presente estudio aportan evidencia científica sobre los cambios en los biomarcadores involucrados en la inmunotoxicidad del arsénico y ofrecen, además, un diseño experimental específico para ensayar potenciales tratamientos frente a la acción deletérea del arsénico trivalente y sus derivados en el sistema inmunitario.
